# A neural glycan HNK-1 is transferred to recipient cells *via* small extracellular vesicles

**DOI:** 10.1016/j.jbc.2026.111144

**Published:** 2026-01-12

**Authors:** Yuko Tokoro, Yasuhiko Kizuka

**Affiliations:** Institute for Glyco-core Research (iGCORE), Gifu University, Gifu, Japan

**Keywords:** extracellular vesicles, glycobiology, glycosylation, glycosyltransferase, *N*-linked glycosylation

## Abstract

Mammalian cells are decorated with a large variety of glycans. Although the biosynthetic enzymes for most glycan structures have been identified, it remains unclear how glycan levels are regulated in cells. Recently, some cellular glycans and their biosynthetic enzymes were found to be loaded into a subset of small extracellular vesicles (sEVs) and transferred to recipient cells, suggesting uncharacterized sEV-mediated mechanisms for glycan remodeling. Here, we found that a brain-specific glycan involved in learning and memory, human natural killer-1 (HNK-1), and its major biosynthetic enzyme, GlcAT-P (B3GAT1), are included in sEVs. Size exclusion chromatography and immunoisolation experiments suggested that the sEVs containing GlcAT-P and glycoproteins with HNK-1 are similar in size but distinct from the tetraspanin-rich sEV subtype. We also found that GlcAT-P in the sEVs is a cleaved form and enzymatically active. Incubation of the HNK-1- and GlcAT-P-loaded sEVs rendered recipient cells positive for HNK-1, whereas sEVs containing GlcAT-P but not HNK-1 did not induce HNK-1 expression in the recipient cells, suggesting that the transfer of HNK-1 but not its biosynthetic enzyme is necessary for recipient cells to be positive for HNK-1. Our findings shed light on a non-genetic pathway for increasing the level of a specific neural glycan *via* sEV-mediated cell–cell communication.

All human cells are decorated with glycans that have a wide variety of structures and play crucial roles in a myriad of biological processes (1, 2). These glycans are expressed in cell type- and protein-selective manners, and the regulated glycan profiles of cells and proteins are essential for their diverse cellular functions (3). More than 7000 proteins are estimated to be *N*-glycosylated (4), but *N*-glycan profiles differ among glycoproteins. Aberrant expression of a specific glycan on proteins sometimes causes the development or exacerbation of various diseases, including cancer, diabetes, and dementia (5, 6, 7). Therefore, it is crucial to fully understand how the level of each glycan is maintained and altered in cells.

Excluding nucleocytosolic *O*-GlcNAc, nearly all glycans are biosynthesized in the endoplasmic reticulum (ER) and the Golgi apparatus by glycosyltransferases (3). From the human genome sequence, approximately 200 glycosyltransferases have been identified, and biochemical analyses have already characterized most of their enzymatic activity (8, 9), enabling us to understand the biosynthetic pathways for almost all mammalian glycans (10). However, glycan biosynthesis in cells depends on multiple factors, including sugar metabolism (11) and intracellular activity and localization of glycosyltransferases (12, 13). In addition, glycoconjugates are degraded and possibly secreted at variable rates, which further contributes to alterations in cellular glycan levels. Therefore, the mechanisms for maintaining the levels of individual glycans in cells are complex and remain to be clarified.

Recently, glycans and their biosynthetic enzymes were found to be present in small extracellular vesicles (sEVs) that are secreted by mammalian cells (14, 15, 16). sEVs contain various biomolecules and can transduce biological signals *via* cell-cell communication. For instance, exosomes, a class of sEVs with a diameter ranging from 50 to 150 nm generated from multivesicular bodies, were reported to be involved in cancer metastasis (17, 18). Glycoconjugates in sEVs were also shown to have a potential impact on the functions of recipient cells and disease pathology. For instance, sEVs from breast cancer cells overexpressing an *N*-glycan branching enzyme, GnT-III (MGAT3), decrease the metastatic ability of recipient cells (19). By contrast, sEVs containing GD2 ganglioside were shown to promote malignant phenotypes in GD2-negative recipient cells (20). Furthermore, we reported that a cancer-promoting *N*-glycan branching enzyme, GnT-V (MGAT5), is selectively enriched in sEVs released from cancer cells, and the uptake of GnT-V-enriched sEVs changed the glycan profiles of the recipient cells to be positive for GnT-V products (14). Similarly, ST6GAL1, a major sialyltransferase for *N*-glycans, was also found to be enriched in smaller extracellular particles called exomeres and suggested to be transferred to recipient cells (21). These findings imply that glycans and glycosyltransferases in sEVs or similar small extracellular particles potentially contribute to functional glycan remodeling in cells, but it is largely unclear what glycans and glycosyltransferases are included in sEVs and whether they are transferred to recipient cells.

Among the various glycan structures, brains express particularly uncommon glycan epitopes, including human natural killer-1 (HNK-1) and polysialic acid (polySia), which are barely expressed in other tissues (22). These neural glycans show highly regulated expression and play crucial roles in neural network formation and synaptic plasticity (22). In addition, their involvement in various diseases, such as neuropathy (23) and schizophrenia (24), indicates that regulation of the expression of brain-specific glycans is essential for maintaining higher-order brain functions. Here, we particularly focus on HNK-1, which we found to be included in sEVs in this study. HNK-1 is a negatively-charged glycan epitope with sulfated glucuronic acid (GlcA) (Fig. 1*A*), and it is present at non-reducing termini of *N*-glycans, *O*-mannose (Man) glycans, and glycolipids (25, 26). HNK-1 is also expressed in some non-neural cell types, such as immune cells (27) and melanoma cells (28), and regulates cell adhesion and migration. As to the biosynthesis of HNK-1, GlcAT-P (B3GAT1) or -S (B3GAT2) and HNK-1ST (CHST10) were identified as responsible enzymes for the sequential transfer of GlcA and sulfate, respectively (29, 30, 31). Knockout (KO) mice lacking the major glucuronyltransferase GlcAT-P showed almost complete loss of HNK-1 in the brain with concomitant impairment in both learning and synapse maturation (32, 33, 34), demonstrating the functional significance of HNK-1 in the brain. However, the mechanisms for maintaining the level of HNK-1 in cells remain unclear.

**Figure 1:**
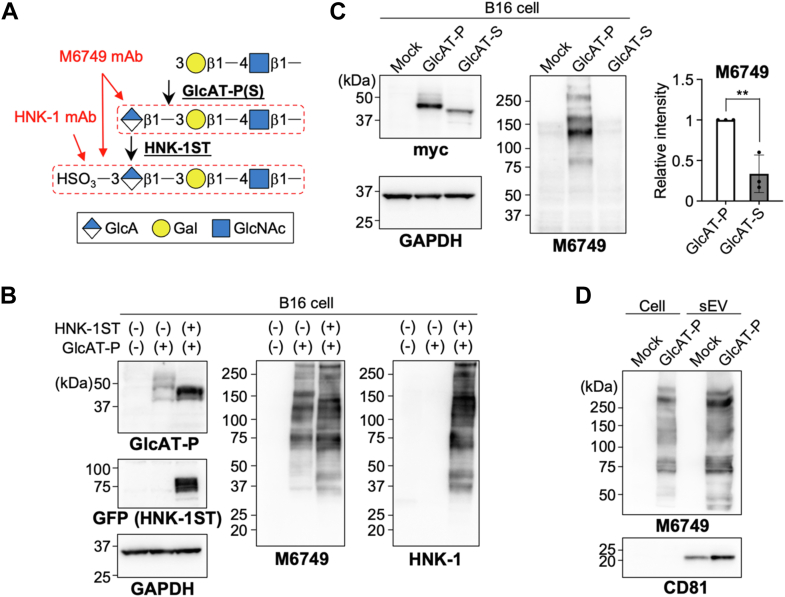
**Expression of neural glyco-epitope HNK-1 in cultured cells and their sEVs.***A*, schematic drawing of HNK-1 biosynthesis and two antibodies that react with HNK-1 and its non-sulfated form. *B*, B16 cells were co-transfected with the plasmids for expressing GlcAT-P and HNK-1ST or the empty vector (−). Cells were lysed and subjected to western blotting with anti-GlcAT-P, anti-GFP, anti-GAPDH, M6749 mAb, and HNK-1 mAb. *C*, B16 cells were transfected with the plasmids for expressing GlcAT-P-myc, GlcAT-S-myc, or the empty vector (mock). Cells were lysed and subjected to Western blotting with anti-myc, anti-GAPDH, and M6749 mAb. The signal intensity of the bands blotted with M6749 was quantified in the right graph (*n* = 3, mean ± SD, ∗∗: *p* < 0.01, unpaired *t* test). *D*, B16 cells were transfected with the plasmid for expressing GlcAT-P or the empty vector (mock). The sEV fractions were collected from the culture media by ultracentrifugation, and the sEV proteins were subjected to western blotting with anti-CD81 and M6749 mAb.

In this study, we explored the possibility that a brain-specific glycan, HNK-1, and its biosynthetic enzyme are packaged into sEVs and transferred to recipient cells. We detected both HNK-1 and GlcAT-P in the sEVs released from parental cells and confirmed that HNK-1 glycans are transferred to recipient cells. Our data revealed an sEV-mediated pathway for remodeling neural glycans, demonstrating the potential of sEVs for the development of a new tool or strategy for modifying specific cellular glycans.

## Results

### Investigation of the presence or absence of HNK-1 glycan in sEVs

To explore whether HNK-1 glycan is included in sEVs, we first expressed GlcAT-P in cultured cells to induce expression of HNK-1. As the endogenous expression and the pathological role of HNK-1 in melanoma cells were reported (35), we used B16 melanoma cells as a model cell system for HNK-1 expression. Although complete biosynthesis of HNK-1 requires sequential transfer of GlcA and sulfate, the non-sulfated form of HNK-1 (nsHNK-1) produced by GlcAT-P or -S can be detected by M6749 mAb (Fig. 1*A*) (36, 37). We confirmed that single expression of GlcAT-P (Uniprot: O35789–1, identical to sGlcAT-P in Fig. 4*B* unless specified) in B16 cells enabled detection with M6749 mAb (Fig. 1*B*), and co-expression of GlcAT-P and HNK-1ST enabled detection with HNK-1 mAb. The downshift of GlcAT-P in SDS-PAGE gel upon co-expression with HNK-1ST was not observed after removing *N*-glycans by peptide *N*-glycanase (PNGaseF) (Fig. S1), suggesting that the band shift was caused by modification of GlcAT-P *N*-glycans by HNK-1ST To simplify the experimental system, hereafter we used single expression of glucuronyltransferase and detection with M6749 mAb. We also compared the levels of nsHNK-1 produced by two glucuronyltransferases GlcAT-P and GlcAT-S in B16 cells. Consistent with the previous *in vitro* study showing that GlcAT-P has the higher enzymatic activity than GlcAT-S (38) and the *in vivo* study showing that HNK-1 glycan almost disappeared in GlcAT-P KO mice (32), expression of GlcAT-P resulted in production of the higher level of nsHNK-1 than that of GlcAT-S in B16 cells (Fig. 1*C*) and in another cell line Neuro2A (Fig. S2*A*), confirming that GlcAT-P is the major glucuronyltransferase for HNK-1 biosynthesis. Based on these results, hereafter we focused on the major HNK-1-synthesizing enzyme GlcAT-P.

Next, we isolated sEVs from the culture medium using an ultracentrifugation method by which exosome-rich sEVs were previously confirmed to be collected from B16 culture medium (14). Western blotting analysis showed that nsHNK-1 was included in sEVs (Fig. 1*D*). Furthermore, we found the similar results showing that nsHNK-1 was included in sEVs derived from Neuro2A cells (Fig. S2*A*) and that endogenous HNK-1 was also included in sEVs released from human melanoma cell Bowes (HMCB) (Fig. S2*B*). Inspired by these findings, we next considered the possibility that nsHNK-1 or its biosynthetic enzyme in sEVs might be transferred from donor cells to recipient cells and be involved in glycan remodeling.

### Presence of GlcAT-P in sEVs

**Figure 2:**
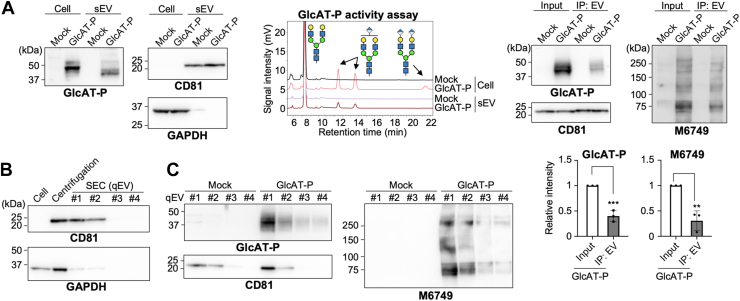
**Presence of GlcAT-P and nsHNK-1 in B16-derived sEVs.***A*, *left* and *middle*, B16 cells were transfected with the plasmid for expressing GlcAT-P or the empty vector (mock). Cells and sEVs from the media were collected, and the proteins were subjected to western blotting with anti-GlcAT-P, anti-CD81, and anti-GAPDH. *Right*, lysates of cells and sEVs were incubated with a fluorescently (PA)-labeled, galactosylated acceptor glycan for GlcAT-P, and the substrate and the products were separated by reversed-phase HPLC. *B*, B16 cells (cell) and their culture media were collected. The sEV fractions were prepared from the media by ultracentrifugation (centrifugation) or by SEC using qEV columns. Proteins in cells and sEV fractions were subjected to western blotting with anti-CD81 and anti-GAPDH. *C*, B16 cells were transfected with the plasmid for expressing GlcAT-P or the empty vector (mock). The sEV fractions were prepared from the media by SEC with qEV columns, and proteins in sEVs were subjected to Western blotting with anti-GlcAT-P, anti-CD81, and M6749 mAb. *D*, B16 cells were transfected with the plasmid for expressing GlcAT-P or the empty vector (mock). sEVs were immunoisolated from the culture media (input) with mixed antibody beads for CD9, CD63, and CD81, and the proteins in immunoprecipitated sEVs were subjected to western blotting with anti-GlcAT-P, anti-CD81, and M6749 mAb. The signal intensity of the bands blotted with anti-GlcAT-P and M6749 was quantified in the *bottom* graphs (*n* = 3, mean ± SD, ∗∗: *p* < 0.01, ∗∗∗: *p* < 0.001, unpaired *t* test).

To test the above possibility, we investigated whether the B16-derived sEVs that contain nsHNK-1 also include GlcAT-P. Western blotting results clearly showed that GlcAT-P is also included in sEVs (Fig. 2*A*). Intriguingly, the observed band size of GlcAT-P in sEVs was smaller than full-length GlcAT-P in cells (Fig. 2*A*, left, second and fourth lanes). This size alteration was found to be generated by N-terminal cleavage as described below (Fig. 4). To examine whether GlcAT-P in sEV is active, we next performed an *in vitro* activity assay for GlcAT-P using cell and sEV extracts as enzyme sources. A fluorescently (pyridylamine, PA)-labeled, galactosylated biantennary glycan was used as an acceptor substrate (39), and the reaction products were separated from the acceptor using reversed-phase HPLC (Fig. 2*A*, right). We were able to clearly detect the GlcAT-P products for the sEV samples as well as for the cell samples, demonstrating that GlcAT-P in sEVs is enzymatically active.

Next, to further characterize the sEVs containing GlcAT-P, we prepared sEVs using two other methods and checked whether GlcAT-P is also included in those sEVs. First, we used qEV35 (pore size of 35 nm) size exclusion chromatography (SEC) columns that were shown to be able to isolate sEVs (40). We confirmed that CD81 (one of tetraspanins)-positive sEVs from B16 cells were enriched in fractions 1 and 2 (Fig. 2*B*), indicating that this technique successfully isolated sEVs based on their size. Furthermore, although GAPDH is known to exist in large EVs (41) and was detected in the centrifuged sample, the levels of GAPDH in the qEV35 samples were very low, indicating the high purity of sEVs prepared by the SEC method. We found that GlcAT-P and nsHNK-1 glycans were also enriched in Fractions 1 and 2 (Fig. 2*C*), demonstrating that GlcAT-P and nsHNK-1 are packaged into sEVs with a similar size range to CD81-positive sEVs. Next, we isolated tetraspanin-rich sEVs by immunoprecipitation (IP) with a mixture of anti-CD9, CD63, and CD81 antibodies and conducted western blotting for GlcAT-P and nsHNK-1. GlcAT-P and nsHNK-1 were detected in the immunoprecipitates, but the enrichment of both GlcAT-P and nsHNK-1 was markedly lower than that of CD81 (Fig. 2*D*). These data suggest that GlcAT-P is contained in sEVs that have similar sizes to the tetraspanin (CD9, CD63, and CD81)-positive sEV subset but are different from sEVs in that subset.

### Direct cell-to-cell transfer of nsHNK-1 glycan *via* sEVs

**Figure 3:**
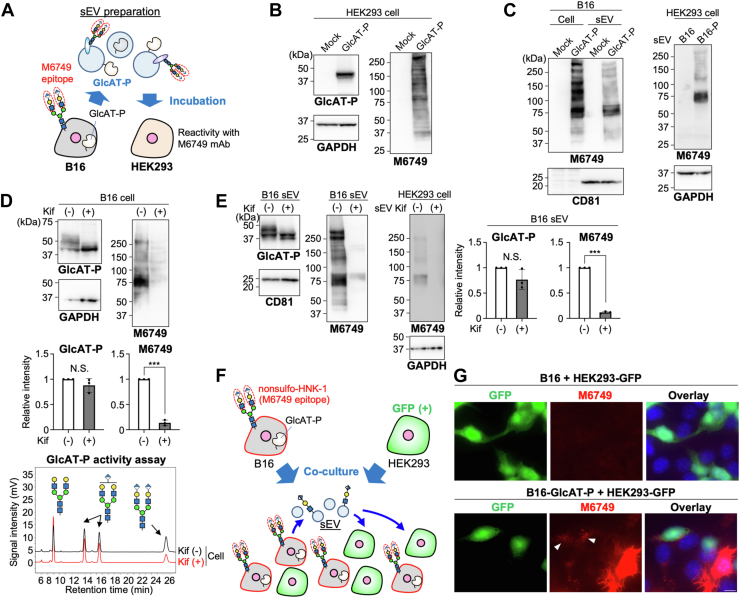
**Direct transfer of sEV-derived nsHNK-1 to recipient cells.***A*, experimental scheme for sEV collection and incorporation. *B*, HEK293 cells were transfected with the plasmid for expressing GlcAT-P or the empty vector (mock). Proteins in HEK293 cells were subjected to western blotting with anti-GlcAT-P, anti-GAPDH, and M6749 mAb. *C*, B16 cells were transfected with the plasmid for expressing GlcAT-P or the empty vector (mock). The sEV fractions were collected from the culture media, and HEK293 cells were incubated with the collected sEVs. Proteins from B16 cells, B16 sEVs, and HEK293 cells were subjected to western blotting with M6749 mAb, anti-CD81, and anti-GAPDH. *D*, *top*, kifunensine (5 μM) was added to the culture medium of B16 cells, and then the cells were transfected with the plasmid for expressing GlcAT-P. Cell lysates were subjected to western blotting with anti-GlcAT-P, anti-GAPDH, and M6749 mAb. Middle, the signal intensity of the bands blotted with anti-GlcAT-P and M6749 was quantified (*n* = 3, mean ± SD, N.S.: not significant, ∗∗∗: *p* < 0.001, unpaired *t* test). *Bottom*, cell lysates were incubated with a PA-labeled, galactosylated acceptor substrate of GlcAT-P, and the substrate and the products were separated by reversed-phase HPLC. *E*, Kifunensine (5 μM) was added to the culture medium of B16 cells, and then the cells were transfected with the plasmid for expressing GlcAT-P. The sEV fraction was collected from the culture medium, and HEK293 cells were incubated with the collected sEVs. Proteins from B16 sEVs and HEK293 cells were subjected to western blotting with anti-GlcAT-P, M6749 mAb, anti-CD81, and anti-GAPDH. The signal intensity of the bands blotted with anti-GlcAT-P and M6749 was quantified in the right graphs (*n* = 3, mean ± SD, N.S.: not significant, ∗∗∗: *p* < 0.001, unpaired *t* test). *F*, experimental scheme for co-culture of B16-GlcAT-P and HEK293-GFP cells. *G*, B16 cells (*upper*) or B16 cells stably expressing GlcAT-P (*lower*) were co-cultured with HEK293 cells stably expressing GFP. Cells were stained with M6749 mAb and DAPI. Scale bar, 10 μm.

We next investigated whether GlcAT-P- and nsHNK-1-containing sEVs can remodel glycans in recipient cells to express nsHNK-1, either by direct transfer of nsHNK-1 glycan or local neo-biosynthesis of nsHNK-1 by transferred GlcAT-P. For this purpose, we isolated sEVs from GlcAT-P-expressing B16 cells and added the sEVs to the culture medium of nsHNK-1-negative HEK293 cells (Fig. 3*A*). We first confirmed that HEK293 cells do not express GlcAT-P and nsHNK-1 endogenously and that exogenous expression of GlcAT-P rendered HEK293 cells reactive with M6749 mAb (Fig. 3*B*). Next, we confirmed that incubation of HEK293 cells with B16-derived sEVs containing GlcAT-P and nsHNK-1 resulted in HEK293 cells that are positive for nsHNK-1 (Fig. 3*C*, right). This indicates that glycan remodeling occurs in the recipient HEK293 cells by incorporating sEVs released from donor cells. We also confirmed that M6749-negative Neuro2A cells became nsHNK-1-positive upon incubation with B16-derived sEVs (Fig. S3).

For sEV-mediated glycan remodeling, we considered two possibilities: one is the direct transfer of nsHNK-1-carrying glycoproteins from sEVs, and the other is neo-biosynthesis of nsHNK-1 in the recipient cells by transfer of active GlcAT-P. To test these possibilities, we prepared sEVs containing active GlcAT-P but not nsHNK-1 using a glycosylation inhibitor. Kifunensine, which inhibits class I mannosidases (42), converts almost all *N*-glycans to immature oligomannose type glycans, which was confirmed by the drastic reduction in the M6749 mAb reactivity of B16 cells even with GlcAT-P overexpression (Fig. 3*D*). Nevertheless, GlcAT-P maintained its enzymatic activity in the presence of kifunensine, as evidenced by the *in vitro* enzyme assay using cell lysates (Fig. 3*D*, chromatogram). We observed that sEVs derived from kifunensine-treated B16 cells expressing GlcAT-P contained a dramatically reduced level of nsHNK-1 but still included GlcAT-P at a comparable level to sEVs from untreated cells (Fig. 3*E*, left and middle panels). Incubation of HEK293 cells with sEVs derived from kifunensine-treated B16 cells expressing GlcAT-P resulted in the loss of nsHNK-1 in the recipient HEK293 cells when compared with the sEVs from untreated B16 cells (Fig. 3*E*, right panel). This strongly suggests that the emergence of nsHNK-1 glycan in the recipient cells is mediated by the direct transfer of nsHNK-1-carrying proteins rather than by the local biosynthesis of nsHNK-1 by transferred GlcAT-P.

To visualize the cell-to-cell transfer of nsHNK-1, we co-cultured B16 cells expressing GlcAT-P and HEK293 cells. To distinguish sEV donor and recipient cells, we used HEK293 cells stably expressing GFP (Fig. 3*F*). In addition, to exclude contamination by the GlcAT-P expression plasmid during transient transfection, we generated stable B16 transfectants expressing GlcAT-P (B16-GlcAT-P) (Fig. S4). Co-culture with B16-GlcAT-P but not with B16 non-transfectants resulted in weak but detectable signals with M6749 mAb in HEK293-GFP (Fig. 3*G*, arrowhead; 13 cells were GFP (+)-M6749 (+) out of 91 GFP (+) cells in 4 independent images acquired using 40x lens). This implies that recipient HEK293-GFP cells acquired nsHNK-1 glycans *via* sEVs released from B16-GlcAT-P cells.

### Efficient sEV-mediated nsHNK-1 transfer by GlcAT-P short form

**Figure 4:**
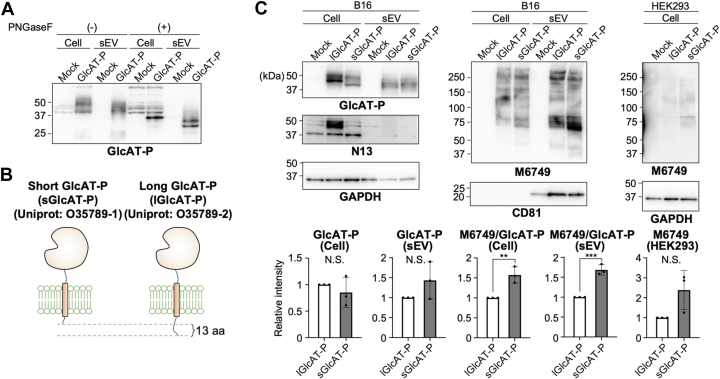
**GlcAT-P in sEV is a truncated form lacking the N-terminal region.***A*, B16 cells were transfected with the plasmid for expressing GlcAT-P or the empty vector (mock), and cells and sEVs from the culture media were collected. Lysates of cells and sEVs were treated with PNGaseF and subjected to western blotting with anti-GlcAT-P. *B*, illustration of short and long forms of GlcAT-P. Rat GlcAT-P isoforms (Uniprot: O35789–1 and −2) were used in this study. *C*, B16 cells were transfected with the plasmids for expressing lGlcAT-P or sGlcAT-P or the empty vector (mock). Cells and sEVs from the culture media were collected, and HEK293 cells were incubated with the collected sEVs. Proteins from B16 cells, B16 sEVs, and HEK293 cells were subjected to western blotting with anti-GlcAT-P, anti-lGlcAT-P-specific N-terminal 13 amino acids (N13), anti-GAPDH, M6749 mAb, and anti-CD81. The signal intensity of the bands blotted with anti-GlcAT-P and M6749 was quantified in the *bottom* graphs (*n* = 3, mean ± SD, N.S.: not significant, ∗∗: *p* < 0.01, ∗∗∗: *p* < 0.001, unpaired *t* test).

We next focused on GlcAT-P in sEVs and examined why GlcAT-P in sEVs is smaller than cellular GlcAT-P in SDS-PAGE (Fig. 2*A*). Because mammalian GlcAT-P has three *N*-glycosylation sites (*e.g.*, Uniprot: O35789–1 for rat GlcAT-P), the difference in size might be derived from different glycan structures between cellular and sEV forms. However, even after removal of *N*-glycans with PNGaseF, GlcAT-P in sEVs is smaller than cellular GlcAT-P (Fig. 4*A*). Considering that GlcAT-P in sEVs is enzymatically active (Fig. 2*A*) and that our previous study showed that GnT-V in sEVs is a soluble form generated by cleavage near the transmembrane border (14), GlcAT-P in sEVs can also undergo proteolysis near the N-terminal region while preserving the intact C-terminal catalytic domain.

We previously reported that mammalian GlcAT-P is expressed as two isoforms (sGlcAT-P and lGlcAT-P) with different lengths of the N-terminal cytosolic tail (Fig. 4*B*) (12). To investigate whether GlcAT-P in sEVs is proteolytically cleaved and whether the N-terminal tail is absent in GlcAT-P in sEVs, we expressed GlcAT-P (sGlcAT-P) and lGlcAT-P in B16 cells and examined their sizes in sEVs. We confirmed that both forms were detected in cells with an anti-catalytic domain antibody (anti-GlcAT-P) (Fig. 4*C*, left top panel), while only lGlcAT-P was recognized by the antibody raised against the lGlcAT-P-specific N-terminal 13 amino acids (N13) (Fig. 4*C*, left middle panel) (12). Notably, both forms of GlcAT-P in sEVs showed identical mobility in an SDS-PAGE gel (Fig. 4*C*, left top panel), and the GlcAT-P in sEVs derived from cells expressing lGlcAT-P was no longer recognized by anti-N13 (Fig. 4*C*, left middle panel). These data show that GlcAT-P in sEVs is a cleaved form lacking the N-terminal region.

We then examined the levels of nsHNK-1 in sEVs. As we reported previously, sGlcAT-P has higher intracellular activity than lGlcAT-P due to its efficient transport from the endoplasmic reticulum to the Golgi apparatus (12). Consistent with this, the levels of nsHNK-1 in sEVs and recipient HEK293 cells were both higher for B16 expressing sGlcAT-P than those expressing lGlcAT-P (Fig. 4*C*, middle and right). These findings indicated that sGlcAT-P leads to more efficient transfer of nsHNK-1 to recipient cells *via* sEVs.

### Involvement of SPPL3 in the packaging of GlcAT-P into sEVs

**Figure 5:**
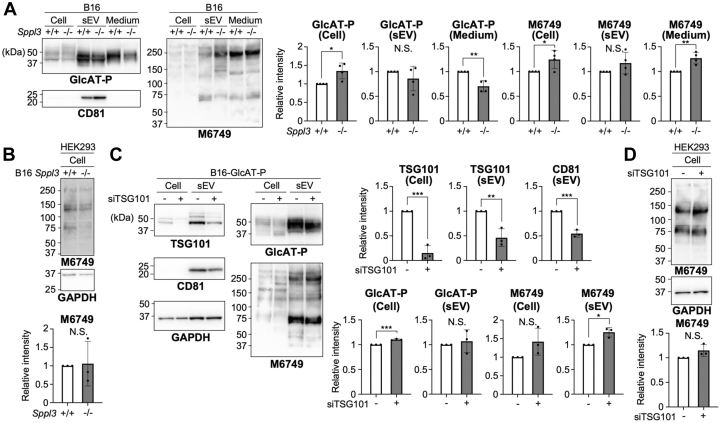
**sEV-mediated transfer of nsHNK-1 in SPPL3-and TSG101-depleted cells.***A* and *B*, B16 WT and SPPL3 KO cells were transfected with the plasmid for expressing GlcAT-P. Cells, sEVs, and the culture media (supernatant after ultracentrifugation) were collected, and HEK293 cells were incubated with the collected sEVs. Proteins from B16 cells, sEVs, and the media were subjected to western blotting with anti-GlcAT-P and M6749 mAb (*A*), and proteins from HEK293 cells were subjected to western blotting with M6749 mAb and anti-GAPDH (*B*). The signal intensity of the bands blotted with anti-GlcAT-P and M6749 was quantified in the graphs (*n* = 4 for A and *n* = 3 for B, mean ± SD, N.S.: not significant, ∗: *p* < 0.05, ∗∗: *p* < 0.01, unpaired *t* test). *C* and *D*, B16 cells stably expressing GlcAT-P were transfected with siRNA targeting TSG101 or a non-targeting siRNA control. Cells and sEVs were collected, and HEK293 cells were incubated with the collected sEVs. Proteins from B16-GlcAT-P cells and sEVs were subjected to western blotting with anti-TSG101, anti-CD81, anti-GAPDH, anti-GlcAT-P, and M6749 mAb (*C*), and proteins from HEK293 cells were subjected to western blotting with M6749 mAb and anti-GAPDH (*D*). The signal intensity of the bands blotted with anti-TSG101, anti-CD81, anti-GlcAT-P, and M6749 was quantified in the graphs (*n* = 3, mean ± SD, N.S.: not significant, ∗: *p* < 0.05, ∗∗: *p* < 0.01, ∗∗∗: *p* < 0.001, unpaired *t* test).

Finally, we explored the loading mechanisms of GlcAT-P and HNK-1 into sEVs. Because GlcAT-P in sEVs is a cleaved form (Fig. 4) and dozens of Golgi-resident glycosyltransferases are primarily cleaved by the membranous protease SPPL3 (43), we investigated the level of GlcAT-P in sEVs and the amount of transferred nsHNK-1 in recipient cells using SPPL3 KO B16 cells which we previously established (44). As expected, the amount of cleaved GlcAT-P in the culture supernatant (after removal of sEVs) was reduced in SPPL3 KO cells (Fig. 5*A*, left upper panel, fifth and sixth lanes), indicating that GlcAT-P cleavage and shedding into the medium are partially dependent on SPPL3, as is the case for many other glycosyltransferases. We also found that the level of GlcAT-P in sEVs tended to be slightly reduced in those from SPPL3 KO cells (Fig. 5*A*, left upper panel, third and fourth lanes). This raises a possibility that the cleavage of GlcAT-P by SPPL3 could contribute to loading in sEVs. However, the levels of transferred nsHNK-1 in the recipient HEK293 cells were comparable between sEVs from wild-type (WT) and SPPL3 KO cells (Fig. 5*B*). This result is consistent with those from kifunensine treatment experiments (Fig. 3), further suggesting that the emergence of nsHNK-1 in recipient cells is derived from the direct transfer of nsHNK-1 glycan *via* sEVs.

TSG101 is a member of the endosomal sorting complex required for transport (ESCRT) machinery and plays a significant role in exosome biogenesis, which is mediated by several pathways involving the ESCRT complex (45) and neutral sphingomyelinase (46). To examine whether the loading of nsHNK-1 into sEVs depends on ESCRT-mediated exosome biogenesis, we depleted TSG101 by siRNA (Fig. 5*C*). The reduced level of CD81 in the sEV fraction from TSG101-knockdown cells confirmed the reduction in exosome release by TSG101 depletion (Fig. 5*C*, left middle panel). By contrast, the levels of GlcAT-P and nsHNK-1 in sEVs were not reduced by TSG101 knockdown. Furthermore, the amount of transferred nsHNK-1 in the recipient HEK293 cells was barely affected by TSG101 knockdown (Fig. 5*D*). These data suggest that exosome biogenesis mediated by ESCRT is not responsible for sEV-mediated transfer of nsHNK-1 into recipient cells.

## Discussion

In this study, we revealed that the non-sulfated form of neural glyco-epitope HNK-1 is transferred from cell to cell *via* sEVs released from donor cells. Furthermore, we found that the responsible enzyme for HNK-1 synthesis, GlcAT-P, is included in sEVs as a cleaved form that maintains its enzymatic activity. Our data suggest that the direct transfer of nsHNK-1-carrying glycoproteins, rather than neo-biosynthesis by transferred GlcAT-P, is the primary mechanism for sEV-mediated glycan remodeling of recipient cells to become nsHNK-1-positive. These findings demonstrate a non-genetic pathway for glycan remodeling of mammalian cells mediated by sEVs.

Currently, the mechanisms for shaping glycan structures in cells have not been fully elucidated. Although it is understood that glycosyltransferases are primarily responsible for biosynthesis of particular glycan structures, their gene expression levels alone cannot fully explain the levels of cellular glycans. One of the gaps between gene expression of glycosyltransferases and the levels of product glycans could be secretion of glycosyltransferases. The presence of many active glycosyltransferases in body fluids was already observed in the 1970s (47), and subsequent studies revealed that a number of Golgi-resident glycosyltransferases are secreted. However, the scarcity of sugar nucleotides in the extracellular fluids (48, 49) led to a long-lasting question about the roles of glycosyltransferases in the extracellular space. We and others previously reported that an *N*-glycan branching enzyme, GnT-V, is shed into culture medium, and the alterations in its shedding significantly impacted the biosynthetic activity of GnT-V in cells (44, 50), suggesting that some glycosyltransferases are discarded from cells into the extracellular fluid to control the cellular enzyme levels. However, recent reports also showed that active GnT-V and ST6GAL1 in sEVs or exomeres are incorporated into recipient cells, potentially contributing to glycan remodeling in recipient cells (14, 21). These findings suggest that glycosyltransferases in sEVs play active roles in glycan remodeling beyond regulating glycosyltransferase activity in their original cells (44).

We showed here that GlcAT-P in sEVs is a proteolytically cleaved form (Fig. 4). Similarly, we previously reported that GnT-V is also present in sEVs as a cleaved form (14). Therefore, the cleavage prior to loading into sEVs might be a common prerequisite for a group of Golgi-resident glycosyltransferases. Furthermore, the cleavage of GlcAT-P partially depends on the SPPL3 protease, which is also responsible for GnT-V cleavage (50). This implies a possible link between SPPL3-mediated cleavage and loading of glycosyltransferases into a subset of sEVs. Since cleaved GnT-V was shown to bind to the outside surface of sEVs *via* the interaction with membrane molecules (14), cleaved GlcAT-P could also reside on the outside surface of sEVs. Notably, enrichment of glycosyltransferases in sEVs is not uniform but rather selective among glycosyltransferases (14). Similarly, SPPL3 does not cleave all Golgi-resident glycosyltransferases (43). To clarify the loading mechanisms and biological roles of glycosyltransferases in sEVs, a more comprehensive analysis will be needed in the future, such as what glycosyltransferases are selectively included in sEVs and whether they are cleaved by SPPL3 or another protease.

Intriguingly, sEVs released from neurons and glial cells regulate brain functions (51, 52). For instance, sEVs produced by cortical neurons altered spine development in neurons (51), and various glycoproteins, including NCAM1, which is a major carrier of the HNK-1 glycan (26), were also found in these sEVs. In that study, HDAC2 transfer was suggested to regulate the sEV-mediated spinogenesis. In another study HNK-1 glycan was also revealed to be involved in spinogenesis (33). Considering these findings, the HNK-1 transfer might be involved in the sEV-mediated regulation of spinogenesis. Furthermore, sEV-mediated glycan remodeling in neural cells was reported to regulate neuronal network synchronicity (53) and release of neurotrophic factors (54). In addition to those physiological functions, there is a need to examine the roles of such sEV-mediated glycan remodeling in the development and aggravation of neurological disorders.

In conclusion, we showed here that the nsHNK-1 glycan epitope is transferred to recipient cells *via* sEVs. An important next step is to elucidate the biological significance of this glycan transfer under physiological and pathological conditions. In addition, deep analysis of the glycan structures of sEVs derived from various cell types as well as the mechanisms for sEV glycan incorporation and glycan remodeling in recipient cells should be clarified in the future. Solving these issues will provide novel clues for understanding the detailed molecular mechanisms of glycan expression in cells, leading to new potential strategies for glycan remodeling for basic science and therapeutics.

## Experimental procedures

### Reagents

The following antibodies were used: mouse anti-GAPDH (Merck Millipore; MAB374), mouse HNK-1 mAb (ATCC; clone Leu7), rabbit anti-FLAG (Cell Signaling Technologies; 14,793), rabbit anti-GFP (MBL; 598), mouse anti-CD81 (Santa Cruz; sc-166029), mouse anti-myc (millipore; 05–724), rabbit anti-TSG101 (abcam; ab125011), HRP-anti-mouse IgG (GE Healthcare; NA931 V), HRP-anti-rabbit IgG (GE Healthcare; NA934 V), HRP-anti-mouse IgM (Invitrogen; 62–6802), Alexa546-anti-rabbit IgG (Invitrogen; A10040), and Alexa488-anti-mouse IgM (Invitrogen; A21042). Rabbit anti-GlcAT-P (GP2) pAb (12) and mouse M6749 mAb (36) were kindly provided by Dr Shogo Oka (Kyoto University). Rabbit pAb against N-terminal 13 amino acids of lGlcAT-P (N13) (12) was generously provided by Dr Jyoji Morise (Kyoto University).

### Plasmid construction

Primers for plasmid construction are listed in Table S1. Construction of pEF-BOS/rat sGlcAT-P (B3GAT1) and pEF-BOS/rat lGlcAT-P was described previously (12). Unless specified, we used pEF-BOS/rat sGlcAT-P for transient expression of GlcAT-P. Construction of p3xFLAG-CMV10/rat GlcAT-P-3xFLAG and pEGFP-N1/rat HNK-1ST (CHST10)-EGFP was described previously (55). For the construction of pcDNA6 mycHisA/rat sGlcAT-P-myc-His, GlcAT-P cDNA was amplified by PCR using pEF-BOS/rat sGlcAT-P as a template and inserted into NotI/XhoI sites. For the construction of pcDNA6 mycHisA/rat GlcAT-S-myc-His, GlcAT-S cDNA was amplified by PCR using pEF-BOS/rat GlcAT-S (30) as a template and inserted into NotI/XhoI sites. For constructing stable transfectants, rat sGlcAT-P cDNA was amplified by PCR using pEF-BOS/rat sGlcAT-P as a template and inserted into EcoRI/NotI sites of a PiggyBac transposon vector (pPBpuro-ErbB1) (56). pPBpuro-ErbB1 was a gift from Dr Michiyuki Matsuda (Addgene plasmid # 197358; http://n2t.net/addgene:197358; RRID:Addgene_197358). pCMV-mPBase (57) was provided by the Sanger Institute.

### Cell culture

HEK293 (ATCC), HEK293-GFP (Cell Biolabs), B16 (RIKEN Cell Bank), B16 SPPL3-KO (14), B16-GlcAT-P, Neuro2A (RIKEN Cell Bank), and HMCB (ATCC) cells were grown at 37 °C under 5% CO_2_ in DMEM supplemented with 10% fetal bovine serum (FBS) and 50 μg/ml kanamycin. For kifunensine treatment, the compound was added to the culture medium at 5 μM followed by 72 h culture. For sEV incubation, sEVs prepared by ultracentrifugation (see “preparation of sEVs”) were suspended in PBS and added to the culture medium (DMEM without FBS) of HEK293 cells followed by 72 h culture. For co-culture experiments, B16-GlcAT-P cells and HEK293-GFP cells were co-cultured (1:1) for 72 h before immunofluorescence staining.

### Transfection of plasmid and siRNA

Cells cultured in a 15-, 10-, or 6-cm dish were transfected with 10, 5, or 2 μg of plasmid using Lipofectamine 3000 Transfection Reagent (Thermo Fisher Scientific) in accordance with the manufacturer’s protocol. For siRNA transfection, B16 cells stably expressing GlcAT-P in a 15-cm dish were transfected with 400 pmol of siRNA targeting mouse TSG101 (Qiagen, SI02694888) or AllStars Negative Control siRNA (Qiagen) using 40 μl of Lipofectamine RNAiMAX Transfection Reagent (Thermo Fisher Scientific) according to the manufacturer’s protocol.

### Establishment of stable transfectants

To generate B16 transfectants stably expressing GlcAT-P with the piggybac transposon system, cells were first co-transfected with pPBpuro/sGlcAT-P and pCMV-mPBase (58). After 24 h of transfection, puromycin was added to the culture medium at 6 μg/ml followed by 24 h culture. Surviving cells were collected and used as stable transfectants after checking the expression of GlcAT-P.

### Western blotting

Cells were solubilized in lysis buffer [TBS containing 1% Nonidet P-40 (NP-40) and protease inhibitor cocktail (Fujifilm)], and sEVs were suspended in PBS (for incubation with recipient cells) or lysis buffer. Protein concentrations were measured using a Pierce BCA Protein Assay (Thermo Fisher Scientific). The samples were mixed with Laemmli sample buffer and subjected to 5%–20% SDS-PAGE and western blotting. Proteins separated by SDS-PAGE were transferred to a nitrocellulose membrane. After blocking with 5% skim milk in TBS containing 0.1% Tween-20 (TBS-T), the membranes were incubated with the primary antibodies overnight at 4 °C. After washing with TBS-T, the membranes were incubated with the HRP-conjugated secondary antibodies for 1 h at room temperature. After washing with TBS-T and TBS, signals were detected with Western Lightning Plus-ECL (PerkinElmer) or SuperSignal West Femto Maximum Sensitivity substrate (Thermo Fisher Scientific). Images were taken using a FUSION-SOLO 7s EDGE (Vilber-Lourmat).

### Immunofluorescence staining

Cells on a glass chamber slide were fixed with PBS containing 4% paraformaldehyde at room temperature for 15 min, washed with PBS three times, and then permeabilized with 0.1% Triton X-100/1% BSA/PBS at room temperature for 15 min. After washing the cells with PBS three times, they were incubated with M6749 mAb for 60 min and then washed with PBS. Alexa546-conjugated secondary antibody and DAPI were added to the cells, followed by further incubation for 30 min. After washing with PBS, cells were mounted with ProLong Diamond Antifade Mountant (Invitrogen). Signals were visualized using a BZ-X800 all-in-one fluorescence microscope (KEYENCE).

### Preparation of sEVs

sEVs were prepared by ultracentrifugation unless specified. We also prepared sEVs using IP or SEC columns (qEV). For ultracentrifugation and IP, we used the same methods as our previous study (14). Briefly, to prepare sEVs by ultracentrifugation, culture medium (two 15-cm dishes) was subjected to 3-step centrifugation and concentration: 1200*g* for 5 min, concentration with Amicon Ultra (cut-off 10 kDa, Millipore) to 500 μl, 3900*g* for 30 min, and 100,000*g* for 60 min. The final pellet was suspended in 100 μl of PBS and used for subsequent experiments (50 μl was used for incorporation experiments against recipient cells). For immunoisolating the tetraspanin-positive sEV subset from the crude sEVs prepared by ultracentrifugation, the EV Isolation Kit Pan mouse (Miltenyi Biotech) was used according to the manufacturer’s protocol. For isolating sEVs by SEC, culture medium (one 15-cm dish) was first subjected to 2-step centrifugation and concentration: 1500*g* for 15 min, concentration with Amicon Ultra (cut-off 10 kDa, Millipore) to 300 μl, and 10,000*g* for 10 min. The supernatant was applied to qEV35original (Meiwafosis) using a qEV automatic fraction collector (AFC, Meiwafosis, 400 μl per each fraction) according to the manufacturer’s protocol. These fractions were ultracentrifuged at 100,000*g* for 60 min, and the resulting pellets were resuspended and used for subsequent experiments. All of the sEV preparations were used for experiments without further characterization.

### GlcAT-P activity assay

Activity of GlcAT-P was measured as described previously (39). Lysates of cells or sEVs were incubated with 10 μl of reaction buffer (125 mM MES, pH 6.2, 10 mM MnCl_2_, 0.2 M GlcNAc, 0.5% TritonX-100, and 1 mg/ml BSA) containing 1 mM UDP-GlcA and 2.5 μM fluorescently labeled acceptor substrate (GGnGGnbi-PA) at 37 °C for 3 h. As a positive control, the recombinant soluble form of proteinA-tagged rat GlcAT-P was used as described previously (59). The reaction was stopped by boiling at 95 °C for 5 min followed by the addition of 40 μl of water. The mixture was centrifuged at 21,500*g* for 5 min, and 10 μl of the supernatant was injected into an HPLC equipped with an ODS column (Inertsil ODS-3, 4.6 × 250 mm; GL Sciences) to detect the acceptor substrate and products. The mobile phase comprised 80% buffer A (20 mM acetate buffer, pH 4.0 adjusted by aqueous ammonia) and 20% buffer B (buffer A containing 1% 1-butanol).

### PNGaseF treatment

For PNGaseF treatment, protein samples were lysed and boiled in Laemmli sample buffer, and PNGaseF (NEB; P0704) or water was directly added to the samples followed by incubation at 37 °C for 2 h. After digestion, the samples were directly used for Western blotting.

### Statistical analysis

Statistical analyses were performed using GraphPad Prism 8 (GraphPad Software). The biological replicates for all experiments were at least 3.

## Data availability

All data are contained within the manuscript.

## Supporting information

This article contains supporting information.

## Conflict of interest

The authors declare that they do not have any conflicts of interest with the content of this article. The author is an Editorial Board Member/Editor-in-Chief/Associate Editor/Guest Editor for this journal and was not involved in the editorial review or the decision to publish this article: One of the authors (Y. K.) is an Editorial Board Member for this journal and was not involved in the editorial review or the decision to publish this article.

## References

[1] Varki A. (2017). Biological roles of glycans. Glycobiology.

[2] Moremen K.W., Tiemeyer M., Nairn A.V. (2012). Vertebrate protein glycosylation: diversity, synthesis and function. Nat. Rev. Mol. Cell Biol..

[3] Schjoldager K.T., Narimatsu Y., Joshi H.J., Clausen H. (2020). Global view of human protein glycosylation pathways and functions. Nat. Rev. Mol. Cell Biol..

[4] Sun S., Hu Y., Ao M., Shah P., Chen J., Yang W. (2019). N-GlycositeAtlas: a database resource for mass spectrometry-based human N-linked glycoprotein and glycosylation site mapping. Clin. Proteomics.

[5] Kizuka Y., Kitazume S., Fujinawa R., Saito T., Iwata N., Saido T.C. (2015). An aberrant sugar modification of BACE1 blocks its lysosomal targeting in Alzheimer's disease. EMBO Mol. Med..

[6] Ohtsubo K., Chen M.Z., Olefsky J.M., Marth J.D. (2011). Pathway to diabetes through attenuation of pancreatic beta cell glycosylation and glucose transport. Nat. Med..

[7] Granovsky M., Fata J., Pawling J., Muller W.J., Khokha R., Dennis J.W. (2000). Suppression of tumor growth and metastasis in Mgat5-deficient mice. Nat. Med..

[8] Moremen K.W., Haltiwanger R.S. (2019). Emerging structural insights into glycosyltransferase-mediated synthesis of glycans. Nat. Chem. Biol..

[9] Narimatsu Y., Bull C., Chen Y.H., Wandall H.H., Yang Z., Clausen H. (2021). Genetic glycoengineering in mammalian cells. J. Biol. Chem..

[10] Huang Y.F., Aoki K., Akase S., Ishihara M., Liu Y.S., Yang G. (2021). Global mapping of glycosylation pathways in human-derived cells. Dev. Cell.

[11] Freeze H.H., Boyce M., Zachara N.E., Hart G.W., Schnaar R.L., Varki A., Cummings R.D., Esko J.D., Stanley P., Hart G.W., Aebi M. (2022). Essentials of Glycobiology.

[12] Kizuka Y., Tonoyama Y., Oka S. (2009). Distinct transport and intracellular activities of two GlcAT-P isoforms. J. Biol. Chem..

[13] Welch L.G., Peak-Chew S.Y., Begum F., Stevens T.J., Munro S. (2021). GOLPH3 and GOLPH3L are broad-spectrum COPI adaptors for sorting into intra-golgi transport vesicles. J. Cell Biol..

[14] Hirata T., Harada Y., Hirosawa K.M., Tokoro Y., Suzuki K.G.N., Kizuka Y. (2023). N-acetylglucosaminyltransferase-V (GnT-V)-enriched small extracellular vesicles mediate N-glycan remodeling in recipient cells. iScience.

[15] Harada Y., Kizuka Y., Tokoro Y., Kondo K., Yagi H., Kato K. (2019). N-glycome inheritance from cells to extracellular vesicles in B16 melanomas. FEBS Lett..

[16] Bienes K.M., Yokoi A., Kitagawa M., Kajiyama H., Thaysen-Andersen M., Kawahara R. (2025). Extracellular vesicles display distinct glycosignatures in high-grade serous ovarian carcinoma. BBA Adv..

[17] Hoshino A., Costa-Silva B., Shen T.L., Rodrigues G., Hashimoto A., Tesic Mark M. (2015). Tumour exosome integrins determine organotropic metastasis. Nature.

[18] Wortzel I., Dror S., Kenific C.M., Lyden D. (2019). Exosome-mediated metastasis: communication from a distance. Dev. Cell.

[19] Tan Z., Cao L., Wu Y., Wang B., Song Z., Yang J. (2020). Bisecting GlcNAc modification diminishes the pro-metastatic functions of small extracellular vesicles from breast cancer cells. J. Extracell Vesicles.

[20] Yesmin F., Furukawa K., Kambe M., Ohmi Y., Bhuiyan R.H., Hasnat M.A. (2023). Extracellular vesicles released from ganglioside GD2-expressing melanoma cells enhance the malignant properties of GD2-negative melanomas. Sci. Rep..

[21] Zhang Q., Higginbotham J.N., Jeppesen D.K., Yang Y.P., Li W., McKinley E.T. (2019). Transfer of functional cargo in exomeres. Cell Rep..

[22] Sytnyk V., Leshchyns'ka I., Schachner M. (2021). Neural glycomics: the sweet side of nervous system functions. Cell Mol. Life Sci..

[23] Delmont E., Attarian S., Antoine J.C., Paul S., Camdessanche J.P., Grapperon A.M. (2019). Relevance of anti-HNK1 antibodies in the management of anti-MAG neuropathies. J. Neurol..

[24] Sato C., Kitajima K. (2021). Polysialylation and disease. Mol. Aspects Med..

[25] Kizuka Y., Oka S. (2012). Regulated expression and neural functions of human natural killer-1 (HNK-1) carbohydrate. Cell Mol. Life Sci..

[26] Morise J., Takematsu H., Oka S. (2017). The role of human natural killer-1 (HNK-1) carbohydrate in neuronal plasticity and disease. Biochim. Biophys. Acta Gen. Subj..

[27] Kared H., Martelli S., Ng T.P., Pender S.L., Larbi A. (2016). CD57 in human natural killer cells and T-lymphocytes. Cancer Immunol. Immunother..

[28] Zamze S., Wing D.R., Wormald M.R., Hunter A.P., Dwek R.A., Harvey D.J. (2001). A family of novel, acidic N-glycans in bowes melanoma tissue plasminogen activator have L2/HNK-1-bearing antennae, many with sulfation of the fucosylated chitobiose core. Eur. J. Biochem..

[29] Terayama K., Oka S., Seiki T., Miki Y., Nakamura A., Kozutsumi Y. (1997). Cloning and functional expression of a novel glucuronyltransferase involved in the biosynthesis of the carbohydrate epitope HNK-1. Proc. Natl. Acad. Sci. U.S.A..

[30] Seiki T., Oka S., Terayama K., Imiya K., Kawasaki T. (1999). Molecular cloning and expression of a second glucuronyltransferase involved in the biosynthesis of the HNK-1 carbohydrate epitope. Biochem. Biophys. Res. Commun..

[31] Bakker H., Friedmann I., Oka S., Kawasaki T., Nifant'ev N., Schachner M. (1997). Expression cloning of a cDNA encoding a sulfotransferase involved in the biosynthesis of the HNK-1 carbohydrate epitope. J. Biol. Chem..

[32] Yamamoto S., Oka S., Inoue M., Shimuta M., Manabe T., Takahashi H. (2002). Mice deficient in nervous system-specific carbohydrate epitope HNK-1 exhibit impaired synaptic plasticity and spatial learning. J. Biol. Chem..

[33] Morita I., Kakuda S., Takeuchi Y., Kawasaki T., Oka S. (2009). HNK-1 (human natural killer-1) glyco-epitope is essential for normal spine morphogenesis in developing hippocampal neurons. Neuroscience.

[34] Morita I., Kakuda S., Takeuchi Y., Itoh S., Kawasaki N., Kizuka Y. (2009). HNK-1 glyco-epitope regulates the stability of the glutamate receptor subunit GluR2 on the neuronal cell surface. J. Biol. Chem..

[35] Casado J.G., Delgado E., Patsavoudi E., Duran E., Sanchez-Correa B., Morgado S. (2008). Functional implications of HNK-1 expression on invasive behaviour of melanoma cells. Tumour Biol..

[36] Obata K., Tanaka H. (1988). Molecular differentiation of the otic vesicle and neural tube in the chick embryo demonstrated by monoclonal antibodies. Neurosci. Res..

[37] Tagawa H., Kizuka Y., Ikeda T., Itoh S., Kawasaki N., Kurihara H. (2005). A non-sulfated form of the HNK-1 carbohydrate is expressed in mouse kidney. J. Biol. Chem..

[38] Kakuda S., Sato Y., Tonoyama Y., Oka S., Kawasaki T. (2005). Different acceptor specificities of two glucuronyltransferases involved in the biosynthesis of HNK-1 carbohydrate. Glycobiology.

[39] Nakano M., Mishra S.K., Tokoro Y., Sato K., Nakajima K., Yamaguchi Y. (2019). Bisecting GlcNAc is a general suppressor of terminal modification of N-glycan. Mol. Cell Proteomics.

[40] Kobayashi H., Shiba T., Yoshida T., Bolidong D., Kato K., Sato Y. (2024). Precise analysis of single small extracellular vesicles using flow cytometry. Sci. Rep..

[41] Visan K.S., Wu L.Y., Voss S., Wuethrich A., Moller A. (2023). Status quo of extracellular vesicle isolation and detection methods for clinical utility. Semin. Cancer Biol..

[42] Elbein A.D., Tropea J.E., Mitchell M., Kaushal G.P. (1990). Kifunensine, a potent inhibitor of the glycoprotein processing mannosidase I. J. Biol. Chem..

[43] Voss M. (2024). Proteolytic cleavage of golgi glycosyltransferases by SPPL3 and other proteases and its implications for cellular glycosylation. Biochim. Biophys. Acta Gen. Subj..

[44] Hirata T., Takata M., Tokoro Y., Nakano M., Kizuka Y. (2022). Shedding of N-acetylglucosaminyltransferase-V is regulated by maturity of cellular N-glycan. Commun. Biol..

[45] Colombo M., Moita C., van Niel G., Kowal J., Vigneron J., Benaroch P. (2013). Analysis of ESCRT functions in exosome biogenesis, composition and secretion highlights the heterogeneity of extracellular vesicles. J. Cell Sci..

[46] Sultana H., Ahmed W., Neelakanta G. (2024). GW4869 inhibitor affects vector competence and tick-borne flavivirus acquisition and transmission by blocking exosome secretion. iScience.

[47] Kim Y.S., Perdomo J., Whitehead J.S. (1972). Glycosyltransferases in human blood.I. galactosyltransferase in human serum and erythrocyte membranes. J. Clin. Invest..

[48] Lazarowski E.R. (2010). Quantification of extracellular UDP-galactose. Anal. Biochem..

[49] Caron P., Nguyen Van Long F., Rouleau M., Bujold E., Fortin P., Mohammadi S. (2022). A liquid chromatography-mass spectrometry assay for the quantification of nucleotide sugars in human plasma and urine specimens and its clinical application. J. Chromatogr. A..

[50] Voss M., Kunzel U., Higel F., Kuhn P.H., Colombo A., Fukumori A. (2014). Shedding of glycan-modifying enzymes by signal peptide peptidase-like 3 (SPPL3) regulates cellular N-glycosylation. EMBO J..

[51] Zhang L., Lin T.V., Yuan Q., Sadoul R., Lam T.T., Bordey A. (2021). Small extracellular vesicles control dendritic spine development through regulation of HDAC2 signaling. J. Neurosci..

[52] Patel M.R., Weaver A.M. (2021). Astrocyte-derived small extracellular vesicles promote synapse formation via fibulin-2-mediated TGF-beta signaling. Cell Rep..

[53] Delaveris C.S., Wang C.L., Riley N.M., Li S., Kulkarni R.U., Bertozzi C.R. (2023). Microglia mediate contact-independent neuronal network remodeling via secreted Neuraminidase-3 associated with extracellular vesicles. ACS Cent. Sci..

[54] Sumida M., Hane M., Yabe U., Shimoda Y., Pearce O.M., Kiso M. (2015). Rapid trimming of cell surface polysialic acid (PolySia) by exovesicular sialidase triggers release of preexisting surface neurotrophin. J. Biol. Chem..

[55] Kizuka Y., Matsui T., Takematsu H., Kozutsumi Y., Kawasaki T., Oka S. (2006). Physical and functional association of glucuronyltransferases and sulfotransferase involved in HNK-1 biosynthesis. J. Biol. Chem..

[56] Matsuda K., Hirayama D., Hino N., Kuno S., Sakaue-Sawano A., Miyawaki A. (2023). Knockout of all ErbB-family genes delineates their roles in proliferation, survival and migration. J. Cell Sci..

[57] Cadinanos J., Bradley A. (2007). Generation of an inducible and optimized piggyBac transposon system. Nucleic Acids Res..

[58] Yusa K., Rad R., Takeda J., Bradley A. (2009). Generation of transgene-free induced pluripotent mouse stem cells by the piggyBac transposon. Nat. Methods.

[59] Kawade H., Morise J., Mishra S.K., Tsujioka S., Oka S., Kizuka Y. (2021). Tissue-specific regulation of HNK-1 biosynthesis by bisecting GlcNAc. Molecules.

