# Supplementary material for: A neural glycan HNK-1 is transferred to recipient cells via small extracellular vesicles
Source: J Biol Chem. 2026 Jan 12;302(2):111144. doi: 10.1016/j.jbc.2026.111144 (PMC12860940; doi:10.1016/j.jbc.2026.111144)
Supplement: Supporting information [file mmc1.pdf]

Supporting Information

HNK-1 glycan is transferred to recipient cells via small extracellular vesicles

Yuko Tokoro, Yasuhiko Kizuka*

*Correspondence: Yasuhiko Kizuka, Ph.D., kizuka.yasuhiko.k8@f.gifu-u.ac.jp

This Supporting information includes:

Fig. S1-4 (included in this PDF)

Table S1 (included in this PDF)

Figure S1

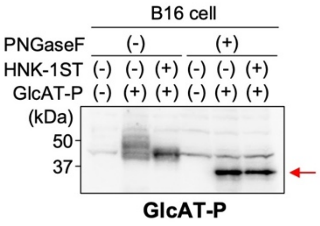

Figure S1. *N*-glycosylation of GlcAT-P expressed in B16 cells. B16 cells were co-transfected with the plasmids for expressing GlcAT-P and HNK-1ST or the empty vector (-). Cells were lysed, treated with PNGaseF, and subjected to western blotting with anti-GlcAT-P.

Figure S2

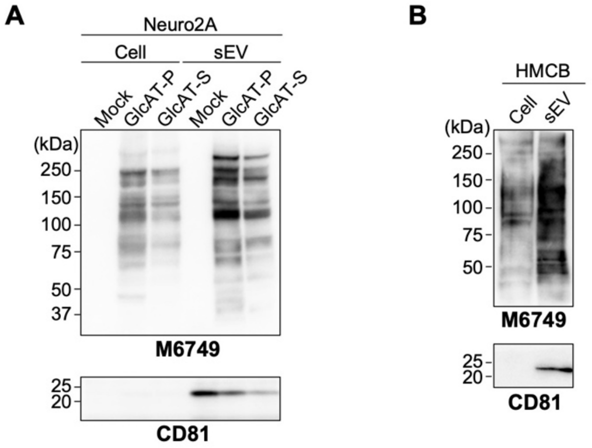

Figure S2. Presence of HNK-1 in Neuro2A- and HMCB-derived sEVs. ***A***, Neuro2A cells were transfected with the plasmids for expressing GlcAT-P-myc, GlcAT-S-myc, or the empty vector (mock). The sEV fractions were collected from the culture media by ultracentrifugation, and the sEV proteins were subjected to western blotting with anti-CD81 and M6749 mAb. ***B***, The sEV fraction was collected from the culture media of HMCB cells by ultracentrifugation, and proteins in the lysates of HMCB cells and sEVs were subjected to western blotting with anti-CD81 and M6749 mAb.

Figure S3

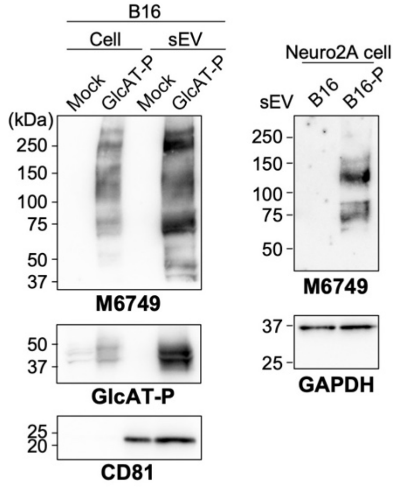

Figure S3. Incorporation of nsHNK-1 in sEVs into Neuro2A cells. B16 cells were transfected with the plasmid for expressing GlcAT-P or the empty vector (mock). The sEV fractions were collected from the culture media, and Neuro2A cells were incubated with the collected sEVs. Proteins from B16 cells, B16 sEVs, and Neuro2A cells were subjected to western blotting with M6749 mAb, anti-GlcAT-P, anti-CD81, and anti-GAPDH.

Figure S4

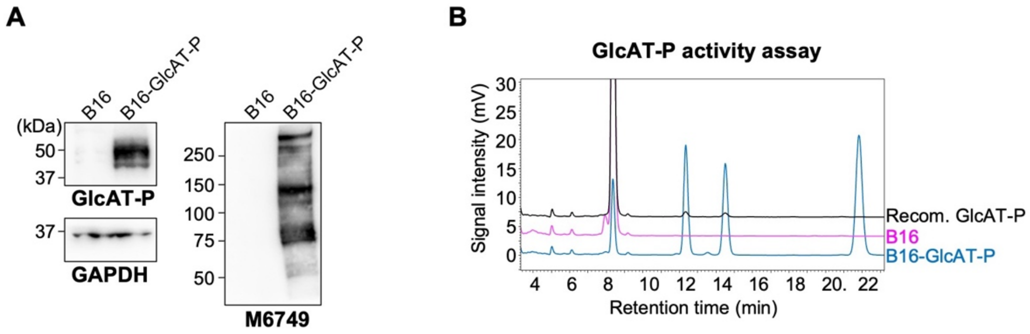

Figure S4. Establishment of B16 stable transfectants expressing GlcAT-P. ***A***, Proteins from WT B16 cells and B16 cells stably expressing GlcAT-P were subjected to western blotting with anti-GlcAT-P, anti-GAPDH, and M6749 mAb. ***B***, Cell lysates were incubated with a PA-labeled, galactosylated acceptor substrate of GlcAT-P, and the substrate and the products were separated by reversed-phase HPLC. Recombinant GlcAT-P was used as a positive control.

Table S1.

Primers used in this study.

Primer_Name	Sequence
rat GlcAT-P-myc-His For	aaaaGCGGCCGCttggagATGCCGAAGAGAAGGGACATC
rat GlcAT-P-myc-His Rev	aaaaCTCGAGGATCTCCACCGAGGGGTCAGTG
rat GlcAT-S-myc-His For	aaaaGCGGCCGCcgcaccATGAAGTCCGCGCTGTG
rat GlcAT-S-myc-His Rev	aaaaCTCGAGCACCTCGATGTTCACTGTGTCCATG
pPBpuro-rat GlcAT-P For	gaattgggatccgaattccgccaccATGCCGAAGAGAAGGGACATC
pPBpuro-rat GlcAT-P Rev	tagttgactctagagcggccTCAGATCTCCACCGAGGGGTCAG
